# PHARC syndrome: an overview

**DOI:** 10.1186/s13023-024-03418-0

**Published:** 2024-11-05

**Authors:** Lusine Harutyunyan, Patrick Callaerts, Sascha Vermeer

**Affiliations:** 1 Laboratory for Behavioral and Developmental Genetics, Department of Human Genetics, KU Leuven, Louvain, Belgium; 2 Centre of Human Genetics, University Hospitals Leuven, Herestraat 49, 3000 Louvain, Belgium; 3 Present Address: Disability Studies, Family Medicine and Population Health, University Antwerp, Antwerp, Belgium

**Keywords:** PHARC, Polyneuropathy, Hearing loss, Cerebellar ataxia, Retinitis pigmentosa, Cataract, Systematic review

## Abstract

PHARC, polyneuropathy, hearing loss, cerebellar ataxia, retinitis pigmentosa and cataracts, or PHARC is a very rare progressive neurodegenerative autosomal recessive disease caused by biallelic mutations in the ABHD12 (a/b-hydrolase domain containing 12) gene, which encodes a lyso-phosphatidylserine (lyso-PS) lipase. The Orpha number for PHARC is ORPHA171848. The clinical picture of PHARC syndrome is very heterogeneous with a wide range of age at onset for each symptom, making a clinical diagnosis very challenging. Differential diagnoses of the disease include Refsum disease, Charcot–Marie–Tooth disease, and Usher syndrome. Many aspects of the disease, such as the biochemistry and pathophysiology, are still not fully understood. We generated a clinical overview of all PHARC patients, including their mutations, described in literature so far. Furthermore, we give an outline of the most recent developments in research on the pathophysiology of PHARC syndrome in an attempt to gain more insight into and increase awareness of the heterogeneity of the disease. We included 58 patients with PHARC from 37 different families with 27 known *ABHD12* mutations. The age at onset (from early childhood to late thirties) and the severity of each feature of PHARC varied widely among patients. Demyelinating polyneuropathy was reported in 91% of the patients. In 86% of patients, hearing loss was present and 74% had cerebellar ataxia, the most variable symptom of PHARC. Retinitis pigmentosa and cataracts occurred in 82% and 86% of patients, respectively. Due to the rareness of the disease and the variable clinical phenotype, a diagnosis of PHARC is often delayed and mostly only made after an extensive genetic work-up. Therefore, we recommend adding the ABHD12 gene to diagnostic gene panels for polyneuropathy, cerebellar ataxia, hearing loss, retinal dystrophy, and cataracts. In addition, a full clinical work-up, neurological (with EMG and neuroimaging of the brain) and ophthalmological (with ERG) examination and audiological tests are indispensable to obtain a comprehensive overview of the clinical phenotype as some symptoms in PHARC may be very subtle and easily overlooked if not tested for. In conclusion, we strongly recommend that patients with (suspected) PHARC should be evaluated in a multidisciplinary setting involving ophthalmologists, audiologists, neurologists, and geneticists to ensure the best possible care. Furthermore, we discuss whether PHARC is a spectrum with various incomplete phenotypes even later in life, or whether it is a syndrome in which the clinical symptoms are variable in severity and age of onset.

## Background

PHARC syndrome, **P**olyneuropathy, **H**earing loss, cerebellar **A**taxia, **R**etinitis pigmentosa, early-onset **C**ataract (MIM 612674), is a rare autosomal recessively inherited, slowly progressive neurodegenerative disease and can represent a complex form of autosomal recessive cerebellar ataxia. It was first described as a Refsum-like disorder in 2009 by Fiskerstrand et al. in a consanguineous Norwegian family with 3 affected family members. The affected members presented with peripheral demyelinating neuropathy, ataxia, spasticity, and pigment retinopathy, later followed by hearing loss and cataracts, which are highly suggestive of Refsum disease. However, they showed normal phytanic and pristanic acid plasma levels as well as a normal enzyme activity for alfa-oxidation [1].

PHARC syndrome is caused by biallelic mutations in the ABHD12 (α/β-hydrolase domain containing 12) gene, which encodes a lyso-phosphatidylserine (lyso-PS) lipase [2]. A suitable animal PHARC model is available, as ABHD12^−/−^ mice exhibit a PHARC-like phenotype [3].

Currently, 58 patients from 37 families have been described with 27 different mutations in the ABHD12 gene, mostly leading to loss of function [1, 2, 4–15]. Most patients exhibit either a homozygous loss of function mutation or are compound heterozygous for a deleterious mutation and a missense mutation. Only two patients had a homozygous missense mutation [6, 9]. The phenotype of these two patients did not show any significant differences compared to the other patients.

PHARC can be considered a spectrum in which the clinical phenotype described in literature ranges from the complete PHARC phenotype to only retinal dystrophy with hearing loss resembling atypical Usher syndrome (USH) [4]. In addition, the age of onset can vary widely (ranging from childhood to late thirties) making a clinical diagnosis of PHARC often difficult. Furthermore, neurological features may develop at a later age which is also true for hearing loss. This means that vigilance for these symptoms is of great importance. A multidisciplinary approach is therefore indispensable.

The exact incidence rates are not known, although a disease incidence of approximately 1/36,000 in Norway was suggested by Fiskerstrand et al. [2].

As of today, no therapy or drug is available to treat or manage PHARC syndrome. Further research is necessary to develop an effective therapy for PHARC patients.

Here, we provide an overview of the patients described in literature and the most recent developments. Our goal is to increase awareness of this rare disease and to gain further insights into this complex syndrome.

### Clinical features and genetics

**Fig. 1:**
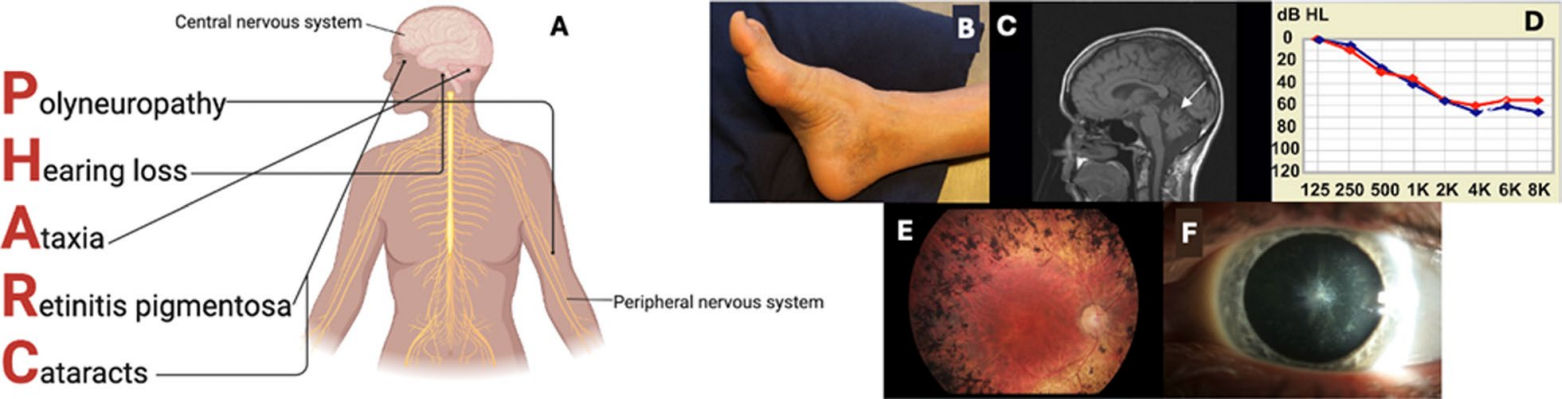
Main symptoms of the PHARC syndrome with observed findings from known PHARC patients. **A** An overview of all clinical symptoms of PHARC syndrome linked to their organ system. **B** Pes cavus and hammer toes can be mild signs of peripheral neuropathy. **C** MRI scan showing cerebellar atrophy (white arrow). **D** Audiogram showing sensorineural hearing loss (SNHL) of both right (red curve) and left (blue curve) ear. **E** Fundus showing bone-spicule-shaped pigment deposits in the mid-periphery, pallor of the optic disc, attenuation of retinal vessels and maculopathy. **F** Star-shaped cataract of the posterior pole of the lens

PHARC is a slowly progressive disease with onset of the first symptoms typically in childhood or in the teens [2]. It involves mostly the central nervous system as well as the peripheral nervous system and the eye. The main features of PHARC include a predominantly demyelinating polyneuropathy, hearing loss, cerebellar ataxia, retinitis pigmentosa and early-onset cataracts, with polyneuropathy as a dominant feature (Fig. 1A). It is important to note that the disease has a wide range of presentations, including severity and order of symptom appearance. Age of onset is highly variable, even within the same family, ranging from childhood to late thirties [8]. To date, no clear phenotype-genotype correlation has been described [6, 11].

#### Neurological features

Demyelinating peripheral polyneuropathy, one of the main features of PHARC, is characterized by damage to the peripheral nerves, resulting in symptoms such as numbness, tingling, weakness, and pain primarily in extremities. Signs of demyelinating polyneuropathy, such as pes cavus and tendoachilles contracture, can be very subtle (Fig. 1B; reused with permission from Fiskerstrand et al. (2010) [2]). In some patients who initially presented with only auditory and visual symptoms, a diagnosis of PHARC syndrome was made after genetic testing. Signs of demyelinating polyneuropathy were only noticed by performing additional neurological investigations with electromyography (EMG) and nerve conduction studies [4]. In other cases, polyneuropathy can be the first indication for a diagnosis of PHARC [8].

Symptoms of cerebellar involvement, including dysarthria, dysmetria, involuntary tremor, ataxic unsteady gait and nystagmus have also been described in PHARC patients [1]. Cerebellar ataxia is the most variable symptom of PHARC and has a variable age of onset. In PHARC, the presentation of cerebellar ataxia can vary from a slight gait abnormality to severe ataxia in all limbs, which presents as a progressive lack of coordination and sometimes involuntary movement of the limbs (2). Dysarthria usually occurs from the first to second decade of life [2]. Magnetic resonance imaging (MRI) or Computational tomography (CT) scans of the brain may reveal cerebellar atrophy (including vermian atrophy), olivoponto-cerebellar atrophy, triventricular hydrocephalus and aqueductal stenosis [2, 11, 16] (Fig. 1C; reused with permission from Fiskerstrand et al. (2010) [2]).

Management of the neurological symptoms of PHARC requires an interdisciplinary approach and generally focuses on symptomatic and supportive care [17]. Some patients with bilateral pes cavus or tendon contracture undergo transfer surgery [5].

#### Hearing loss

Progressive sensorineural hearing loss (SNHL), ranging from moderate to severe, is a very common symptom in PHARC patients and its onset is often at a young age [18]. SNHL refers to damage to the inner ear or auditory nerve pathways that transmit sound to the brain. This results in difficulties in hearing and understanding sounds [19] (Fig. 1D; reused with permission from Fiskerstrand et al. (2010) [2]). Auditory function is assessed by pure tone audiometry, speech audiometry and tympanometry. Many PHARC patients will eventually use a hearing aid or undergo cochlear implant surgery [4].

#### Ophthalmological features

Retinitis pigmentosa, present in most PHARC patients, is characterized by progressive degeneration of photosensitive cells in the retina and typically occurs between the second and third decades with distinctive electroretinogram (ERG) abnormalities indicative of rod-cone dystrophy [2] (Fig. 1E; reused with permission from Fiskerstrand et al. (2010) [2]). Rod photoreceptor cells are responsible for vision under low-light conditions and for peripheral vision. As these cells are usually first affected, most patients with PHARC syndrome have a history of nyctalopia (night blindness) in the early phase of the disease. Visual symptoms may progressively decline, which can eventually lead to severe visual impairment [6]. Multimodal imaging techniques, including fundoscopy and full-field ERG, are necessary for establishing a correct ophthalmological diagnosis since rod-cone dystrophy can easily be overlooked [14].

Additionally, cataracts are a common feature of PHARC syndrome and are assessed with a visual acuity test and a slit-lamp examination [14]. Cataracts are characterized by clouding of the crystalline lens and may progress over time (Fig. 1F). Individuals may experience blurred vision, decreased visual acuity, sensitivity to bright light and difficulty with glare [20]. Nguyen et al. (2021) described distinct types of cataracts, including congenital cataracts, in patients diagnosed with PHARC. Many PHARC patients receive cataract surgery (13).

#### Differential diagnosis

PHARC syndrome may present with heterogeneous clinical symptoms with very different ages of onset, making a diagnosis very challenging. PHARC syndrome should always be in the differential diagnosis in patients who are clinically suspected of having Refsum disease [1], Charcot–Marie–Tooth syndrome (CMT) [10] or Usher syndrome [4, 7]. In particular, in patients suspected of having Usher syndrome, the most common cause of deafness and blindness [7, 21], that could not be genetically confirmed, PHARC should be included in the differential diagnosis. Furthermore, a thorough neurological investigation should be performed, as well as drawing a pedigree, for a correct and fast diagnosis of PHARC syndrome [11]. Notably, *ABHD12* mutations were identified by whole-exome sequencing (WES) in patients who were clinically diagnosed with Usher syndrome type 3. These patients showed hearing loss and reduced visual acuity. However, there was no record of neurological investigation with the distinct possibility that subtle signs of polyneuropathy and ataxia were overlooked [22]. Furthermore, genetic testing in patients with a so-called isolated form of Usher syndrome characterized by peripheral and macular retinal disease revealed biallelic mutations in *ABHD12*, without records of neurological investigations or neuroimaging as well [13].

### ABHD12 gene and mutations

PHARC is caused by biallelic mutations in the ABHD12 gene. The gene encompasses 13 exons and is located on chromosome 20p11.21. Different transcripts are described of which the longest and main transcript contains all 13 exons (NM_001042472.3) [6]. *ABHD12* expression is abundant in the thyroid and the brain, with a particularly high expression in the cerebellum and many other tissues [3, 23]. In mice, *ABHD12* expression is also detected in the lens [24].

From the different ABHD12 mutations described in literature, the most common pathogenic variant is c.337_338delGAinsTTT in exon 3, which was first described in a Norwegian family by Fiskerstrand et al. and fully segregated with the disease in this large family. It is suggested that this mutation is a common European founder mutation [1, 2]. This mutation causes a frameshift of the reading frame with a downstream premature stop codon, which results in a loss of function of *ABHD12* when assessed with activity-based protein profiling (ABPP) [5]. Other mutation types include nonsense, missense and splice site mutations. No genotype–phenotype correlation has been established thus far [14]. Two patients with a homozygous missense mutation have been described, with one showing the full clinical picture of PHARC from the age of 7 years [9].

### ABHD12 protein and function

The ABHD12 gene encodes a ~ 45 kDa glycoprotein single-pass integral membrane serine hydrolase of 398 amino acids [25]. The ABHD12 protein is significantly enriched in the microsomal fraction (> 90%) of mouse brain cells and mammalian cells, which consists primarily of the endoplasmic reticulum [26]. ABHD12 is a member of the serine hydrolase family, which also includes ABHD6, ABHD16A and monoacylglycerol lipase (MAGL) [25, 27]. ABHD12 contains an α/β-hydrolase domain fold and catalyzes the hydrolysis of (lyso-)PS [3, 23], ox-PS [28] and monoacylglycerols in vivo, including the endocannabinoid 2-arachidonoylglycerol (2-AG) [25, 26, 29].

The activity of ABHD12 was first studied by Blankman et al. in 2007 in the microglial cell line BV-2 using ABPP. Previously uncharacterized ABHD6 and ABHD12 were identified and found to have the same activity as MAGL. MAGL, ABHD6 and ABHD12 collectively contribute to about 98% of 2-AG hydrolysis into arachidonic acid (AA) and glycerol in the brain [25]. A minor contribution (~ 1%) is made by fatty acid amide hydrolase (FAAH), which will not be discussed in further detail. Each of the first three mentioned hydrolases exhibit heterogeneous cellular and subcellular distributions, which results in distinct 2-AG pools in the brain [23, 25, 26]. Compared to MAGL, which is responsible for over 85% of 2-AG degradation in the brain, specifically in microglia, ABHD12 hydrolyzes only a small fraction of 2-AG [25, 30].

The 27 different pathogenic variants in *ABHD12* identified in PHARC patients have all been predicted to lead to a significant loss of lipase activity [1, 2, 4–15]. Based on the hydrolysis of 2-AG by ABHD12, it was proposed that disruption of the endocannabinoid signaling system was central to the phenotypes observed in PHARC patients and a mouse model [2]. However, given the minor contribution of ABDH12 to 2-AG hydrolysis, 2-AG is unlikely to be the cause of the phenotypic changes linked to PHARC syndrome. Furthermore, ABHD12^−/−^ mice lack the typical clinical signs attributed to increased signaling via CB1, such as hypothermia, hypomotility, or analgesia [2]. This triggered researchers to look for another ABHD12 ligand responsible for the metabolic and neurobehavioral phenotypes (auditory and motor defects) of PHARC syndrome [3].

### Lyso-phosphatidylserine and pathophysiology of PHARC

The deletion of *ABHD12* in mice had no significant effect on the brain 2-AG levels, which implies that ABHD12 metabolizes another lipid and that PHARC is probably not caused by a defect in the endocannabinoid pathway. Comparative mass spectrometry-based untargeted lipidomics [31] was performed on the brains of wild-type and ABHD12^−/−^ mice to map the biological pathways regulated by ABHD12 in vivo. ABHD12^−/−^ mice exhibit a PHARC-like phenotype and are therefore considered a suitable animal PHARC model. Lipidomic analysis revealed that the deletion of ABHD12 resulted in the accumulation of a non-endocannabinoid metabolite called lyso-PS and that ABHD12 efficiently hydrolyzed lyso-PS in vitro [3]. The lyso-PS hydrolase activity of ABHD12 was also demonstrated in a study in which HEK293T cells transfected with murine ABHD12 showed increased hydrolytic activity toward multiple lysophospholipids. In addition, ABHD12^−/−^ brain membrane homogenates and lymphoblast cell lines derived from PHARC patients, displayed decreased lyso-PS lipase activity and increased levels of lyso-PS [3, 23]. Very long chain lyso-PS (VLC-lyso-PS), C20:4 lyso-PS, C20:4 PS, phosphatidylglycerol and lysophosphatidylinositol lipid levels are significantly increased in ABHD12^−/−^ mouse brains [3].

**Fig. 2:**
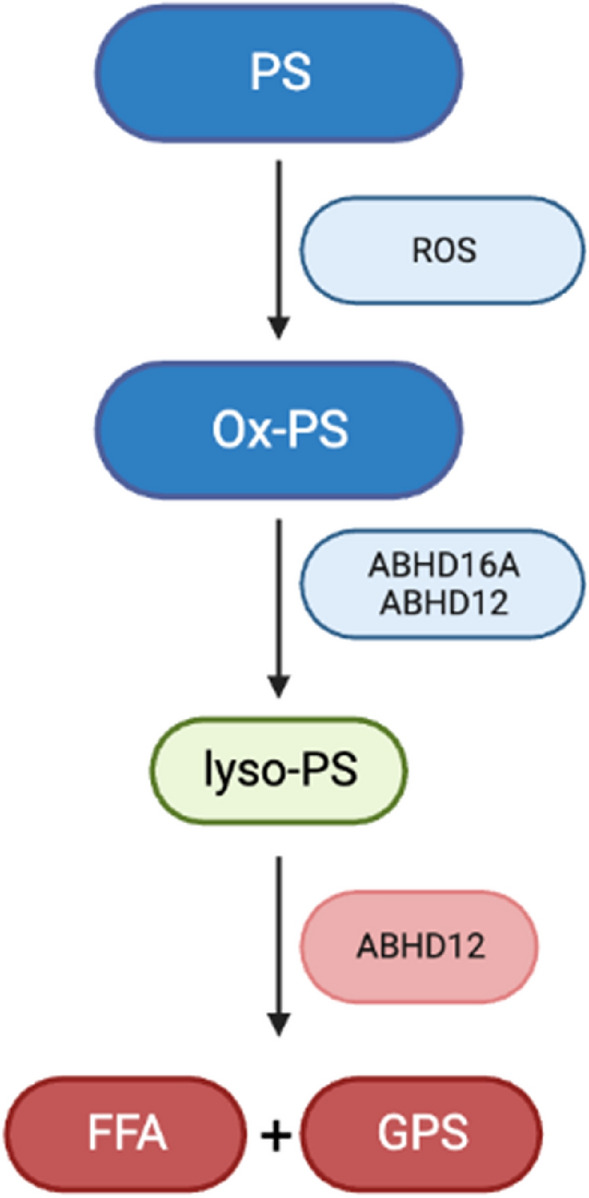
Synthesis and degradation of lyso-PS in the mouse brain. PS = phosphatidylserine; ROS = reactive-oxygen species; ox-PS = oxidized phosphatidylserine; FFA = free fatty acid; GPS = glycerophosphoserine

Intracellular lyso-PS is synthesized through ox-PS hydrolysis by ABHD16A, with a minor contribution from ABHD12, in mammalian cells and in vivo [23, 28] (Fig. 2). Phosphatidylserine-specific phospholipase (PS-PLA1) also shows PS lipase activity but is reported to be a secreted enzyme and therefore unlikely to have access to the majority of PS lipids in cells [32]. ABHD16A and ABHD12 are responsible for maintaining the levels of ox-PS lipids under oxidative stress [28, 33]. The interplay between these enzymes regulates immunological processes by creating a lipid signaling network, that is dynamically controlled by and contributes to the macrophage inflammatory response [23].

**Fig. 3:**
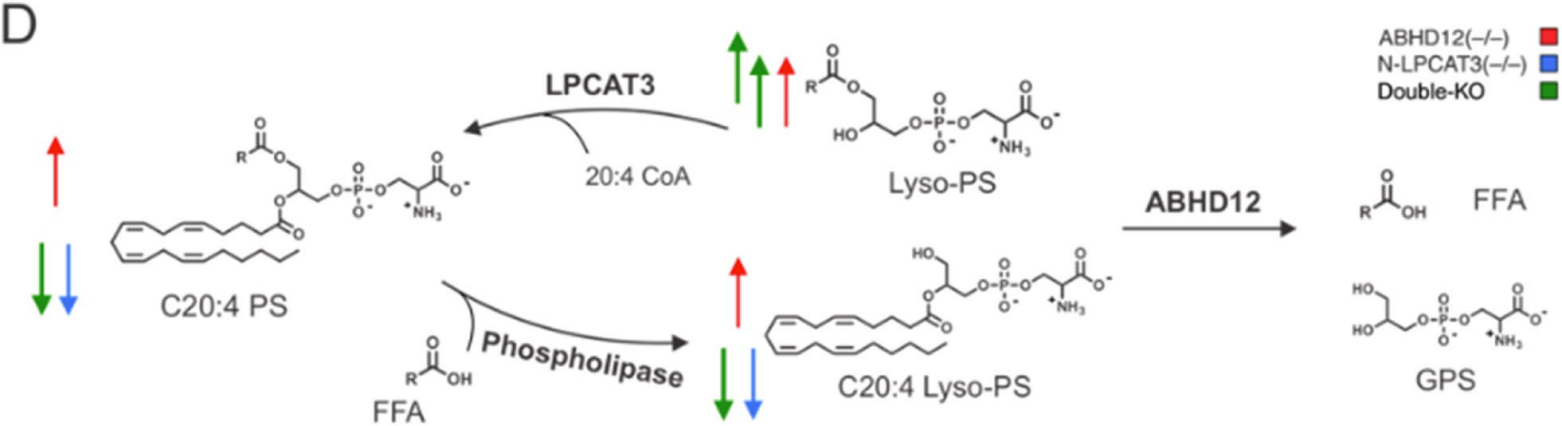
Double knockout of ABHD12 and LPCAT3 causes a strong increase in lyso-PS, but not in C20:4 PS and C20:4 lyso-PS. Metabolic pathway diagram illustrating the coordinated regulation of lyso-PS and C20:4 PS levels in the mammalian brain by ABHD12 and LPCAT3. Red arrows indicate the change in lipid content in ABHD12^−/−^ mice, blue arrows indicate the change in lipid content in N-LPCAT3^−/−^ mice and green arrows indicate the change in lipid content in double knockout mice. FFA = free fatty acid, GPS = glycerophosphoserine

The interaction between ABHD12 and lysophospholipid acetyltransferase (LPCAT3), which converts lyso-PS to C20:4 PS, has been reported, such that genetic or pharmacological disruption of ABHD12 causes LPCAT3 to act as a rate-limiting enzyme to control the lyso-PS and C20:4 PS concentrations. These enzymes coordinately control the lyso-PS and C20:4 PS levels in the mammalian brain as the lipid profile of LPCAT3 and ABHD12 double-knockout mice resembled that of LPCAT3^−/−^ mice. As a result of the rate-limiting role of LPCAT3, knocking out ABHD12 in the mouse brain caused an increase in both lyso-PS levels and C20:4 PS levels (Fig. 3; reused with permission from Ichu et al. (2020). Copyright 2020, American Chemical Society [34]). The question remained which lipid was responsible for the neuropathological phenotypes in ABHD12^−/−^ mice. Compared with ABHD12^−/−^ mice, ABHD12 and LPCAT3 double-knockout mice exhibited a hyperincrease in lyso-PS and a decrease in C20:4 PS and displayed greater loss of auditory function and increased activity of microglia and macrophages (Fig. 3). These findings suggest that the increase in the lyso-PS level, rather than in the C20:4 PS level, is the cause of the microgliosis and auditory dysfunction in ABHD12^−/−^ mice [34].

Lyso-PS is a key player in several immunological and neurological responses and is reported to be abundant in the central nervous system, primary mouse immune cells and primary human lymphoblast cell lines [3, 5, 23].

**Fig. 4:**
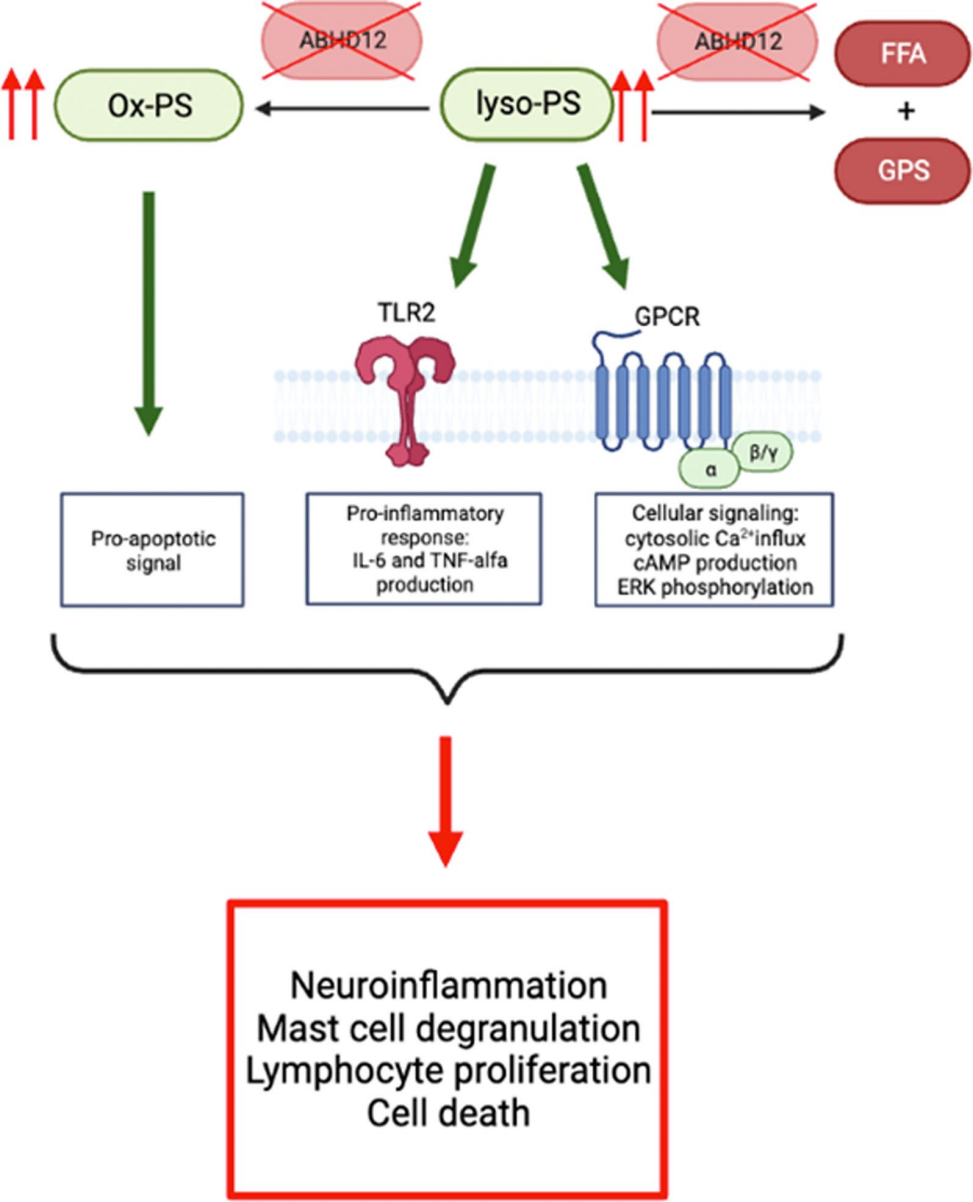
Lyso-PS and ox-PS may play key roles in the pathophysiology of PHARC syndrome in ABHD12^−/−^ mice. Silencing of ABHD12 results in the accumulation of lyso-PS and ox-PS, which results in a proinflammatory response and cellular signaling by lyso-PS through its receptors and a proapoptotic signal by ox-PS. These cellular changes result in neuroinflammation, mast cell degranulation, lymphocyte proliferation and cell death, which are likely to be the underlying mechanisms of the pathophysiology of PHARC syndrome. TLR2 = toll-like receptor 2; GPCR = G-protein-coupled receptor; FFA = free fatty acid; GPS = glycerophosphoserine

In ABHD12^−/−^ mice, a prominent increase in lyso-PS and ox-PS levels in the brain occurred prior to the onset of neuroinflammatory and behavioral defects, suggesting that these signaling lipids are the cause of the observed PHARC-like symptoms [3, 28] (Fig. 4). Silencing ABHD12 also results in the accumulation of the signaling lipids lyso-PS and ox-PS in mammalian cells [23, 28]. Ox-PS levels are elevated as a result of increased reactive oxygen species (ROS), with ox-PS generating an apoptotic signal that is recognized by phagocytes [35].

Lyso-PS induces immunological and neurological responses that include mast cell degranulation [36, 37], modulation of the phagocytic activity of macrophages, increased cytokine secretion by immune cells, lymphoblast stimulation [23], among others [33] (Fig. 4). Several studies have revealed that lyso-PS plays a role in both the induction and resolution of acute inflammatory responses [38]. Furthermore, Ogasawara et al. showed that pharmacological ABHD12 disruption causes elevated cytokine production [39, 40].

The best characterized function of lyso-PS is the stimulation of mast cell degranulation in rat peritoneal cells [36, 37]. When administered intravenously in rodents, the induction of anaphylaxis, hyperglycemia in the brain and hypothermia was also observed, because of systemic histamine release in the blood stream [41, 42]. Furthermore, an increase in the cellular levels of lyso-PS in microglial BV-2 cell lines and primary microglia stimulates lipopolysaccharide-induced phagocytic activity, which suggests that lyso-PS plays an important role in the exacerbation of neuroinflammation in activated microglia [43]. Phagocytosis of apoptotic cells by macrophages is also called efferocytosis and is established via the presence of lyso-PS on apoptotic neutrophils [38]. This implies that lyso-PS plays a major role in the resolution of inflammation and the restoration of cell and tissue function [44].

Lyso-PS activates different G-protein coupled receptors (GPCRs), among which GPR34 [45], P2Y10 [46] and GPR174 [47]. These GPCRs are also called lyso-PS receptors. To understand why ABHD12 knockout causes all the different physiological impairments observed in PHARC, it is crucial to identify the interactions of lyso-PS and the functions of the lyso-PS receptors.

GPR34 is highly expressed in human mast cells and can induce a major histamine release upon activation by lyso-PS [45], exacerbating neuropathic pain by inducing a proinflammatory microglial response. GPR34 antagonists have been found to soothe this pain [48]. Nonetheless, the exact role of GPR34 is still under investigation since knocking-out this receptor provided no evidence for it being the receptor for lyso-PS nor did it affect mast cell degranulation and migration [49]. The least characterized lyso-PS receptor, P2Y10, also a putative lyso-PS receptor expressed in different immune cells, including mast cells [33, 46], and functional compensation of P2Y10 may be the reason why GPR34 knockout mast cells in mice show no modulation of the inflammatory response [50].

Lyso-PS can also suppress naive T-cell proliferation and IL-2 production in vivo by activating GPCR34 and GPCR174, respectively, which are abundantly expressed in microglia and lymphoid organs [51–53].

Blankman et al. [3] hypothesized that the Toll-like receptor 2 (TLR2) might be activated by VLC-lyso-PS, which was identified in ABHD12^−/−^ mice (23). TLR2 is upregulated on dendritic cells after microglial activation [54]. Overstimulation of TLR2 signaling regulates microglial phagocytosis of neurons, initiating a neuroinflammatory response and eventually cell death, in primary neural cell cultures upon viral infection [55] and in vivo [3, 40, 56, 57]. Neuroinflammation is a significant feature of PHARC, suggesting that TLR2 might be involved in PHARC pathophysiology [58].

## Methods

A comprehensive literature review was conducted. PubMed was utilized as a resource to collect all relevant information regarding the PHARC syndrome. The following keywords were used in our search: ‘PHARC’, ‘ABHD12’, ‘polyneuropathy hearing loss ataxia retinitis pigmentosa cataracts’ and ‘lysophosphatidylserine’. All clinical information of the described patients with PHARC syndrome and all known mutations associated with the disease to date was compiled and is presented in Tables 1 and 2.

**Table 1:** 58 patients with PHARC syndrome from 37 different families have been described in literature to our knowledge

					Polyneuropathy, cerebellar ataxia				Hearing loss		Retinitis pigmentosa		Cataract		Other	
Reference	Family	Patient (sex/age at first examination or diagnosis in years)	Country	Age at onset (years)	Neuropathy	Age at onset (years)	Ataxia	Age at onset (years)	Deafness	Age at onset (years)	Retinitis pigmentosa/Night blindness	Age at onset (years)	Cataract	Age at onset (years)	Other symptoms	Age at onset (years)
Fiskerstrand et al. [1]	I.1	♀, 59	Norway	Early twenties	Demyelinating sensory motor polyneuropathy	38	N	/	Sensorineural	29	Y	38	Y	28	Foot deformities	Childhood
	I.2	♂, 53	Norway	37	Demyelinating polyneuropathy	37	Y	37	Sensorineural	Thirties	Y	37	Y	49	Pes cavus	Childhood
	I.3	♂, 43	Norway	Childhood	Demyelinating sensory motor polyneuropathy	38	Y	43	Y	Childhood	Y	46	Y	Twenties	Cerebellar atrophy	/
Fiskerstrand et al. [10]	II.1	♂, 58	Norway	Teens	Demyelinating and axonal polyneuropathy	51	N	/	Y	Twenties	Y	35	Y	25	Pes cavus	NK
	II.2	♀, 54	Norway	Teens	Demyelinating neuropathy	53	N	/	Y	Twenties	Y	25	Y	32	Pes cavus	NK
	III	♀, 36	Norway	Teens	Demyelinating neuropathy	NK	Y	NK	Y	10	Y	36	Y	15	Pes cavus	NK
	IV	♂, 24	Norway	Teens	Demyelinating neuropathy	NK	N	/	Y	Late in teens	N	/	Y	16	Pes cavus; hammer toes	NK
	V	♂, 16	Norway	Teens	Demyelinating neuropathy	NK	N	/	Y	13	N	/	Y	NK	Pes cavus	NK
	VI.1	♂, 11	Algeria	3–4	Demyelinating neuropathy	NK	Y	3–4	N	/	N	/	N	/	N	NK
	VI.2	♀, 10	Algeria	4–5	Demyelinating neuropathy	NK	Y	4–5	N	/	N	/	N	/	N	NK
	VII.1	♂, 44	Algeria	7–10	Demyelinating neuropathy	NK	Y	7–10	Y	NK	N	/	NK	/	Pes cavus	NK
	VII.2	♀, 26	Algeria	4–9	Severe demyelinating sensory motor polyneuropathy	NK	Y	4–9	Y	NK	Y	NK	Y	NK	Pes cavus	NK
	VIII.1	♀, 26	Algeria	6	Severe demyelinating sensory motor polyneuropathy	NK	Y	6–12	Y	6	N	/	N	/	Pes cavus	NK
	VIII.2	♀, 19	Algeria	NK	Demyelinating neuropathy	12	N	/	NK	/	NK	/	NK	/	Pes cavus	19
	IX	♀, 32	Algeria	16–20	Axonal polyneuropathy	NK	Y	16–20	Y	NK	N	/	N	/	Pes cavus	NK
	X	♀, 50	USA	Teens	Sensory neuropathy	34	Y	18	Y	17	Y	Twenties	Y	22	Pes cavus	34
	XI.1	♂, 24	UAE	Childhood	Motor neuropathy	Childhood	Y	NK	Y	14	Y	Twenties	Y	15	Pes cavus	NK
	XI.2	♂, 20	UAE	2	Demyelinating neuropathy	4	Y	2	Y	6	Y	NK	Y	NK	Pes cavus	NK
	XI.3	♀, 6	UAE	< =6	Demyelinating neuropathy	NK	Y	NK	Y	NK	N	/	Y	NK	N	/
Eisenberger et al. [4]	XII.1	♀, 55	Lebanon	17	N	/	Y	19	Progressive severe to profound sensorineural	17	Y	18	Y	26	Atrophy of macular region Glaucoma Esotropia	40 55 55
	XII.2	♂, 53	Lebanon	18	N	/	N	/	Progressive severe to profound sensorineural	24	Y	18	Y	23	Atrophy of macular region Glaucoma	50
Chen et al. [5]	XIII	♀, 29	USA	3–5	Demyelinating sensory motor neuropathy	27	Y	NK	Progressive severe sensorineural	Early childhood	Y	22	Y	21	Bilateral foot deformities Macular edema Mild dysmetria	At birth 22 NK
Nishiguchi et al. [6]	XIV.1	♀, 78	Spain	NK	N	/	N	/	Sensorineural	NK	Y	38	Y	NK	N	/
	XIV.2	♂, 75	Spain	NK	N	/	N	/	Severe sensorineural	NK	Y	Thirties	Y	NK	Cholesteatoma	NK
	XIV.3	♀, 72	Spain	NK	N	/	N	/	N	/	Y	Thirties	Y	NK	N	/
	XIV.4	♂, 66	Spain	NK	N	/	N	/	Sensorineural	NK	Y	Thirties	Y	NK	N	/
	XV	♀, 38	Netherlands	NK	Demyelinating sensory motor neuropathy	38	N	/	Sensorineural	Twenties	Y	NK	Y	21	N	/
	XVI	♂, 34	Netherlands	NK	Distal sensory neuropathy	NK	Y	35	Moderate sensorineural	Thirties	Y	NK	Y	29	N	/
Yoshimura et al. [7]	XVII.1	♂, 64	Japan	Early childhood	NK	NK	Y	63	Progressive profound sensorineural	< 10	Y	45	Y	45	Epilepsy Cerebellar atrophy	NK
	XVII.2	♂, NK	Japan	NK	NK	NK	Y	27	Y	NK	Y	NK	NK	18	Epilepsy	NK
	XVII.3	♂, NK	Japan	NK	NK	NK	NK	NK	Y	NK	Y	NK	N	/	NK	NK
	XVIII	♂, 56	Japan	15	Peripheral neuropathy	NK	Y	27	Progressive severe sensorineural	15	Y	22	Y	18	N	/
Bek-Tol et al. [8]	XIX.1	♂, 31	Netherlands	Childhood	Demyelinating polyneuropathy	NK	Y	Childhood	Y	20	NK	NK	Y	NK	Achilles contractures Cerebellar atrophy	Childhood NK
	XIX.2	♂, 25	Netherlands	Childhood	Demyelinating polyneuropathy	NK	Y	NK	Y	NK	Y	Childhood	Y	NK	N	/
	XIX.3	♂, 27	Netherlands	Childhood	Demyelinating polyneuropathy	NK	N	/	N	/	Y	NK	Y	Childhood	N	/
Tingaud-Sequeira et al. [9]	XX	♀, 31	Sweden	7	Demyelinating sensory motor neuropathy	15–16	Y	16	Progressive profound sensorineural	7	Y	16	Y	8	N	/
Lerat et al. [10]	XXI	♂, 36	France	5	Sensory motor polyneuropathy	15	Y	15	Sensorineural	5	N	/	Y	28	N	/
Frasquet et al. [15]	XXII.1	♂, 41	Spain	Childhood	Demyelinating polyneuropathy	12	Y	NK	Sensorineural	30	Y	NK	Y	NK	Foot deformities Cerebellar atrophy	Childhood NK
	XXII.2	♂, NK	Spain	Childhood	Demyelinating polyneuropathy	17	Y	NK	Sensorineural	18–20	Y	40	Y	30	Foot deformities Dysarthria, dysmetria Cerebellar atrophy	Childhood NK NK
Thimm et al. [11]	XXIII.1	♂, 23	Iraq	9	Demyelinating sensory motor neuropathy	NK	Y	NK	Y	9	N	/	Y	NK	Bilateral pes cavus Cerebellar atrophy	NK 22
	XXIII.2	♂, 27	Iraq	12	Demyelinating sensory motor neuropathy	NK	Y	NK	Y	12	Y	NK	Y	NK	NK	NK
Igelman et al. [13]	XXIV	♀, 48	NK	18	NK	NK	NK	NK	N	/	Y	NK	Y	NK	Atrophy in macular region	NK
	XXV	♀, 23	NK	20	NK	NK	NK	NK	N	/	Y	NK	N	NK	Atrophy in macular region	NK
	XXVI	♂, 17	NK	16	NK	NK	NK	NK	N	/	Y	NK	N	NK	Bull's eye maculopathy	NK
	XXVII	♂, 27	NK	22	NK	NK	NK	NK	Moderate progressive	20	Y	NK	Y	NK	Atrophy in macular region	NK
	XXVIII	♂, 42	NK	Early 30 s	NK	NK	NK	NK	Moderate progressive	44	Y	NK	Y	NK	Hole in macular region	NK
	XXIX	♂, 53	NK	18	NK	NK	NK	NK	Severe progressive	20	Y	NK	N	NK	Atrophy in macular region	NK
Dias Bastos et al. [12]	XXX	♀, 29	Portugal	16	Demyelinating sensory motor neuropathy	NK	Y	NK	Y	16	Y	19	Y	19	Low stature Pes cavus Cerebellar atrophy	NK
Nguyen et al. [14]	XXXI	♂, 47	NK	8	Y	8	NK	8	Y	28	Y	45	Y	36	N	/
	XXXII	♀, 32	NK	Childhood	Y	Childhood	NK	45	Y	17	Y	32	Y	32	N	/
	XXXIII.1	♂, 33	NK	3	Demyelinating neuropathy	27	NK	27	N	/	Y	14	Y	3	Macular atrophy	NK
	XXXIII.2	♂, 33	NK	3	Severe demyelinating polyneuropathy	Childhood	NK	Childhood	Y	NK	Y	21	Y	3	Macular atrophy	NK
	XXXIII.3	♂, 38	NK	4	Severe demyelinating polyneuropathy	Childhood	Y	Childhood	Y	20	Y	NK	Y	4	Macular atrophy Cerebellar atrophy	NK
	XXXIV	♀, 36	NK	NK	NK	NK	NK	NK	Y	12	Y	31	Y	32	N	NK
	XXXV.1	♂, 20	Belgium	16	Demyelinating sensory motor neuropathy	20	N	/	Moderate sensorineural	16	Y	16	Y	17	Macular atrophy Cerebellar atrophy	NK
	XXXV.2	♂, 17	Belgium	10	Demyelinating sensory motor neuropathy	18	Y	NK	Moderate sensorineural	10	Y	10	Y	10	Macular atrophy Cerebellar atrophy	NK
	XXXVI	♀, 46	NK	NK	Y	47	Y	47	Y	NK	Y	NK	Y	NK	Macular atrophy	NK
	XXXVII	♂, 39	NK	NK	NK	NK	NK	NK	Y	33	Y	23	Y	29	Macular atrophy	NK

Overview of all PHARC patients with their symptoms and ages at onset Related patients are indicated with the same Roman numeral. Early childhood is defined as three years old, childhood as 8 years old, and teens as 15 years old

*Y*, yes; *N* no; *NK*, not known

*Deducted from own data

**Table 2:** 27 different ABHD12 mutations known to cause PHARC syndrome are described in literature to our knowledge to date

Reference	Family	Country	Mutation type	Homozygous	Nucleotide change	Amino acid change	Localization	Domain
Fiskerstrand et al. [1]	I	Norway	Frameshift	Homozygous	c.337_338delGAinsTTT	p.Asp113Phefs*15	exon 3	EC
Fiskerstrand et al. [2]	II, III, IV, V	Norway	Frameshift	Homozygous	c.337_338delGAinsTTT	p.Asp113Phefs*15	exon 3	EC
	VI, VII, VIII, IX	Algeria	Frameshift	Homozygous	c.846_852dupTAAGAGC	p.His285fs*1	exon 9	EC
	X	USA	Nonsense	Homozygous	c.1054C>T	p.Arg352*	exon 12	EC
	XI	UAE	Large deletion	Homozygous	14 kb deletion including exon 1	p.?	exon 1	Cytoplasmic
Eisenberger et al. [4]	XII	Lebanon	Nonsense	Homozygous	c.193C>T	p.Arg65*	exon 2	Cytoplasmic
Chen et al. [5]	XIII	USA	Nonsense	Compound heterozygous	c.1129A>T	p.Lys377*	exon 12	EC
			Large deletion		59 kb deletion including exon 1	p.?	exon 1	Cytoplasmic
Nishiguchi et al. [6]	XIV	Spain	Frameshift	Compound heterozygous	c.319delA	p.Arg107Glufs*8	exon 3	EC
			Missense		c.605C>T	p.Thr202Ile	exon 6	NK
	XV	Netherlands	Missense	Homozygous	c.1116C>G	p.His372Gln	exon 12	EC
	XVI	Netherlands	Nonsense	Compound heterozygous	c.477G>A	p.Trp159*	exon 4	EC
			Missense		c.557G>C	p.Arg186Pro	exon 5	EC
Yoshimura et al. [7]	XVII, XVIII	Japan	Splice-altering	Homozygous	c.316+2T>A	p.?	intron 2	Cytoplasmic
Bek-Tol et al. [8]	XIX	Netherlands	Frameshift	Compound heterozygous	c.337_338delGAinsTTT	p.Asp113Phefs*15	exon 3	EC
			Splice-altering		c.423-1_425del	p.Trp141_His142delinsCys	exon 4	EC
Tingaud-Sequeira et al. [9]	XX	Sweden	Missense	Homozygous	c.758G>C	p.Thr253Arg	exon 8	EC
Lerat et al. [10]	XXI	France	Frameshift	Homozygous	c.379_385delAACTACTinsGATTCCTTATATAC-CATTGTAGTCTTACT-GCTTTTGGTGAA-CACA	p.Asn127Aspfs*23	exon 3	EC
Frasquet et al. [15]	XXII	Spain	Frameshift	Homozygous	c.211_223del	p.Arg71Tyrfs*26	exon 2	EC
Thimm et al. [11]	XXIII	Iraq	Nonsense	Homozygous	c.784C>T	p.Arg262*	exon 8	EC
Igelman et al. [13]	XXIV	NK	Nonsense	Compound heterozygous	c.1054C>T	p.Arg352*	exon 12	EC
			Frameshift		c.1196del	p.*399Serfs*122		?
	XXV	NK	Nonsense	Compound heterozygous	c.1063C>T	p.Arg355*	exon 12	?
			Missense		c.259C>A	p.Pro87Thr	exon 2	?
	XXVI	NK	Nonsense	Homozygous	c.193C>T	p.Arg65*	exon 2	Cytoplasmic
	XXVII	NK	Splice-altering	Homozygous	c.620-2A>G	p.?	intron 6	?
	XXVIII	NK	Missense	Compound heterozygous	c.374C>T	p.Thr125Met	exon 3	?
			Missense		c.1154T>C	p.Leu385Pro	exon 12	?
	XXIX	NK	Nonsense	Compound heterozygous	c.784C>T	p.Arg262*	exon 8	EC
			Splice-altering		c.867+5G>A	p.?	intron 9	?
Dias Bastos et al. [12]	XXX	Portugal	Nonsense	Homozygous	c.1054C>T	p.Arg352*	exon 12	EC
Nguyen et al. [14]	XXXI	NK	Frameshift	Compound heterozygous	c.337_338delGAinsTTT	p.Asp113Phefs*15	exon 3	EC
			Frameshift		c.1075del	p.Val359Phefs*27	exon 12	?
	XXXII	NK	Frameshift	Homozygous	c.337_338delGAinsTTT	p.Asp113Phefs*15	exon 3	EC
	XXXIII	NK	Frameshift	Compound heterozygous	c.337_338delGAinsTTT	p.Asp113Phefs*15	exon 3	EC
			Splice-altering		c.423-1_425del	p.Trp141_His142delinsCys	exon 4	EC
	XXXIV	NK	Frameshift	Homozygous	c.337_338delGAinsTTT	p.Asp113Phefs*15	exon 3	EC
	XXXV	Belgium	Frameshift	Homozygous	c.337_338delGAinsTTT	p.Asp113Phefs*15	exon 3	EC
	XXXVI	NK	Nonsense	Homozygous	c.1063C>T	p.Arg355*	exon 12	NK
	XXXVII	NK	Frameshift	Compound heterozygous	c.337_338delGAinsTTT	p.Asp113Phefs*15	exon 3	EC
					c.341dup	p.Leu114Phefs*14	exon 3	NK

Overview of all pathogenic variants of ABHD12 that cause PHARC syndrome

*EC*, extracellular

## Results

### Clinical phenotype

To provide a phenotypic overview of all patients, we surveyed the available literature and presented all the data in Table 1.

Among 58 patients with PHARC that are described thus far, only 20 patients (34%) exhibited all the main features of the disease with a mean age of 37 years, ranging from 6 to 78 years old. The mean age at onset of the first symptom of PHARC was 11 years old (ranging from 2 to 37 years). The first signs can be subtle symptoms, such as polyneuropathy, but hearing or visual symptoms can also be very mild in the early stages of the disease and may therefore not be a recognizable feature [1, 5].

Polyneuropathy was reported in 91% (N = 41/45) of patients who underwent neurological examination with EMG, and in 71% (N = 41/58) of all patients, described in literature thus far. The mean age of onset was approximately 23 years (ranging from childhood to 53 years) (Table 1).

Cerebellar ataxia is the most variable symptom of PHARC syndrome, with a very wide range of ages of onset (2–63 years). Approximately 71% of the described patients with PHARC syndrome (N = 35/49) who underwent neurological examination, had cerebellar ataxia, with a mean age at onset of 21 years (ranging from two to 45 years) (Table 1). Among all 58 patients, only 60% showed signs of cerebellar ataxia. However, not all patients underwent an MRI or CT scan. Cerebellar atrophy was mentioned in almost half of the patients who underwent an MRI or CT scan (N = 20/41).

In 49 of the 57 investigated patients (86%) SNHL or complete deafness was detected, with a mean age at onset of approximately 16 years, ranging from early childhood up to 44 years old (Table 1). Auditory examination was performed in almost all patients, as hearing loss can be one of the first symptoms of PHARC syndrome.

Retinitis pigmentosa occurred in 47 out of 56 (84%) patients who underwent ophthalmological investigations, and in 81% (N = 47/58) of all reported patients, with a mean age at onset of 18 years. The age at onset ranges from childhood until 45 years of age (Table 1). Fundus examination may reveal macular atrophy and changes in the retinal pigment epithelium and intraretinal specular hyperpigmentation has been demonstrated in some patients [14].

Visually significant cataracts are usually observed between the second and fourth decades of life but can also be observed in children. The mean age at onset was 22 years and described in 86% (N = 47/55) of the PHARC patients who were ophthalmologically investigated and in 81% (N = 47/58) of all patients (Table 1).

### ABHD12 mutations

**Fig. 5:**
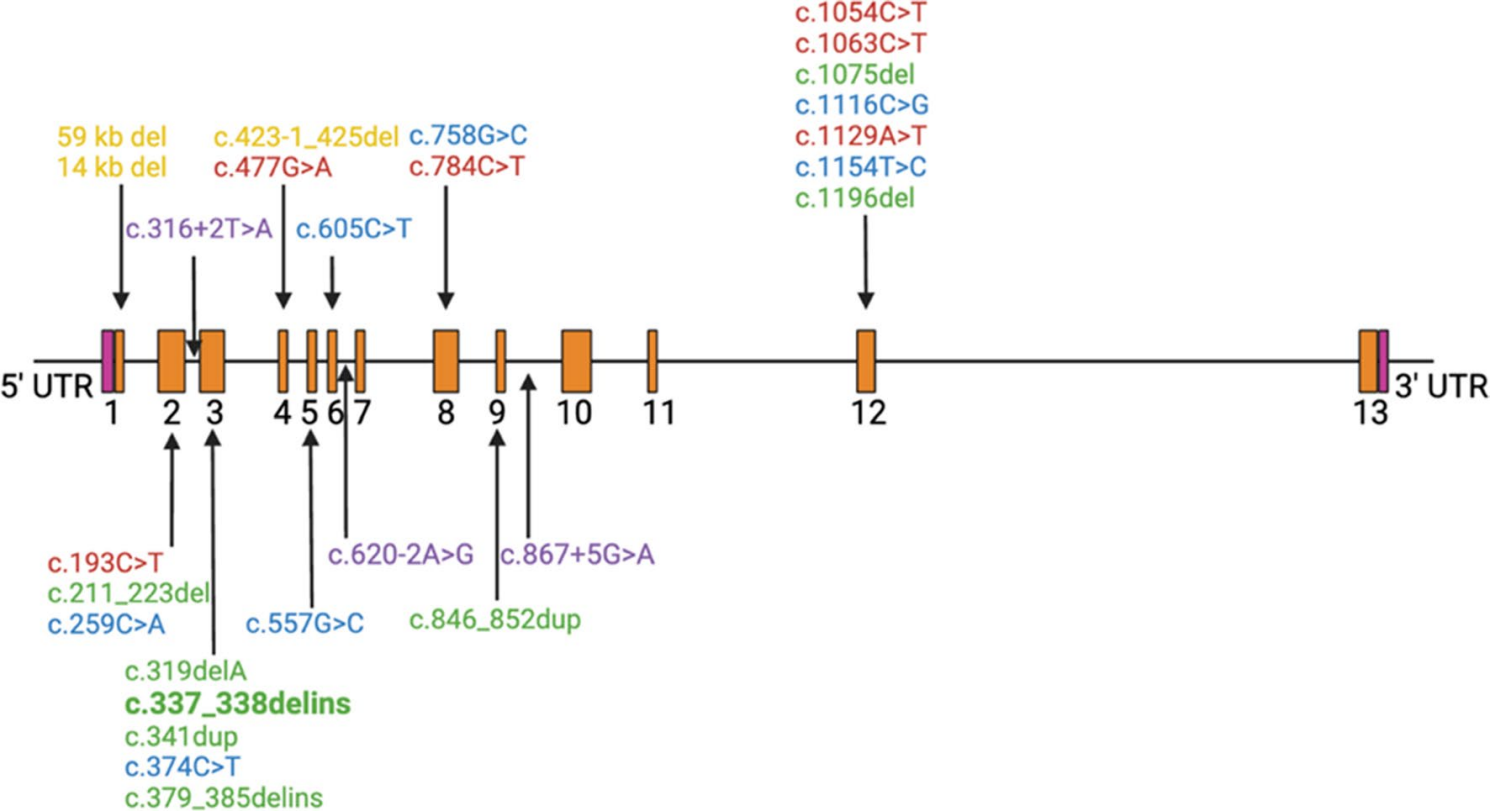
Overview of the different known ABHD12 gene mutations. The exons are indicated in orange: nonsense mutations (red), frameshift mutations (green), missense mutations (blue), deletions (yellow) and splice-altering mutations (purple). c.337_338delinsGAinsTTT (in bold) is the most common mutation

The different ABHD12 mutations that cause PHARC syndrome described in literature are summarized in Table 2 in order of appearance and linked to the families from Table 1. Twenty-seven different pathogenic ABHD12 variants associated with PHARC syndrome have been identified thus far, with the most common pathogenic variant being c.337_338delGAinsTTT (~ 32%; 12 out of 37 families) in exon 3, which predicts the replacement of an asparagine at codon 113 with phenylalanine and a frameshift mutation (p.Asp113Phefs*15). This mutation was first described by Fiskerstrand et al. (2010) in the Norwegian patients and is widespread among European patients, suggesting a common ancestor [1, 2]. Exon three and exon 12, in which most mutations (12 of 27) are located, seem to be more prone to pathogenic variants (Fig. 5). Almost all mutations were confirmed or predicted to have a loss of function effect on ABHD12, apart from a small deletion in intron 6 in family XXVII and a splice-altering mutations in intron 9 in family XXIX and in intron 2 detected in families XVII and XVIII (Table 2). The large deletions in families XI and XIII, result in the complete absence of proteins [2, 5].

## Discussion

In this systematic review, we describe all patients with PHARC syndrome and associated *ABDH12* mutations so far reported in literature, to gain more insight into the clinical phenotype and the pathophysiological mechanisms, with the aim of better understanding the course of the disease.

Currently, 58 patients from 37 families with biallelic mutations in the ABHD12 gene, involved in PHARC have been described [1, 2, 4–14]. The full clinical picture of PHARC consists of polyneuropathy, with SNHL, cerebellar ataxia (often with pyramidal tract signs such as spasticity, hyperreflexia and extensor plantar response), retinal dystrophy (rode cone dystrophy and macular dystrophy), and cataracts. The clinical picture of PHARC is very heterogeneous, ranging from the full clinical spectrum to isolated retinal dystrophy (with or without hearing loss) with an age of onset between 2 and 37 years (Table 1). The mean age of onset of PHARC was only 11 years old (Table 1). Of all patients described in literature (n = 58), only 20 patients showed the full clinical picture of PHARC syndrome. The incomplete clinical picture of a large proportion of patients (28%), is mostly due to incomplete phenotypic assessment, the young age at examination and/or the absence of follow-up, which results in missing phenotypic data.

Our clinical overview (Table 1) shows that a demyelinating polyneuropathy is one of the most common features of PHARC, as up to 91% of patients who underwent neurological investigation with an EMG and up to 71% of all PHARC patients have a polyneuropathy. The age at onset ranged from childhood years to 53 years old, with a mean age of 23 years. Mild signs of polyneuropathy, such as pes cavus, were observed in 19 patients and are usually present at birth or in early childhood. Due to missing EMG recordings in 13 cases [4, 7, 13], it cannot be ruled out that polyneuropathy might be a consistent feature of PHARC when performing an EMG in every PHARC patient. Symptoms of numbness, prickling or tingling can be very subtle or even absent at the beginning of a polyneuropathy. Interestingly, this is supported by all 19 patients described by Fiskerstrand et al. (2009 and 2010) who exhibited signs of polyneuropathy. For instance, Nishiguchi et al. (2014) reported that none of the 4 siblings from family XIV had polyneuropathy (Table 1) with a mean age of 72 years (ranging from 66 to 78 years), but as there were no records of EMG, it might be possible that a mild form of polyneuropathy was overlooked [6].

Cerebellar ataxia was quite common, as 60% of all patients or 71% of the 49 patients who underwent neurological examination and/or imaging studies showed signs of cerebellar ataxia. For the other 9 patients, it is not known whether they had any signs of ataxia, as there were no records of neurological examination or neuroimaging [4, 7, 13, 14]. The age of the patients who did show signs of ataxia ranged from as young as 2 to 63 years old, which makes this feature the most variable feature of PHARC syndrome. Brain scans of many patients with ataxia revealed complementary cerebellar atrophy, with some specifically in the vermis of the cerebellum [2].

Hearing loss ranging from mild to severe (leading to deafness) is also a variable but quite common feature of PHARC syndrome. SNHL typically develops gradually and may worsen over time. It occurred in about 49 patients (86%) who underwent auditory investigation (n = 57).

Another important clinical feature of PHARC is retinitis pigmentosa, which is typically diagnosed in the second or third decade of life. It occurred in 84% (47/56) of the ophthalmologically investigated patients with PHARC and in 81% of all described patients.

The last and common feature of PHARC is cataract, which occurs in 86% of the patients who were ophthalmologically investigated, with a mean age of onset of 22 years (range 3–49 years) (Table 1).

One of the main features of PHARC are signs of polyneuropathy, such as numbness, prickling or tingling in the feet (or hands), pes cavus, hammer toes and foot drop, which can be very subtle and easily overlooked in the early stages of the disease. Additionally, hearing loss and decreased visual acuity, are symptoms of PHARC, which can be very mild and can be easily missed in the initial phase of the disease. In some patients, hearing loss and a decrease in visual acuity are diagnosed during early childhood.

Polyneuropathy, the main feature of PHARC can present in the early stages of the disease with sometimes subtle signs of numbness, a prickling or tingling feeling in the feet (or hands), or a mild pes cavus which can be easily overlooked. As these clinical signs might easily be missed, a multidisciplinary approach involving a neurologist, an ophthalmologist, and an ear-nose-throat (ENT) physician is crucial for obtaining reliable clinical data about the phenotypic spectrum of PHARC syndrome. Furthermore, a yearly follow-up is recommended. This entails looking for signs of mild neuropathy that might go unnoticed in patients with retinopathy and hearing loss [7], as well as ophthalmological evaluation of patients presenting with polyneuropathy and hearing impairment [15]. For instance, in the publication by Igelman et al. (2021), patients with biallelic *ABHD12* mutations were diagnosed with atypical Usher syndrome, as they showed retinal dystrophy without SNHL. However, there are no records of any neurological examinations, EMGs, or imaging tests, which can lead to missing out on subtle neurological symptoms. As the authors state, a long-term follow-up is required to conclusively determine whether this is truly atypical Usher syndrome or eventually PHARC syndrome [13].

In addition, identifying biallelic mutations in *ABHD12* is of great importance to confirm the clinical diagnosis of PHARC syndrome. This literature review reveals the heterogeneous nature of PHARC syndrome, and the variable age of onset of the symptoms. Therefore, we recommend adding the ABHD12 gene to diagnostic gene panels for hearing loss, retinal dystrophy, cataracts, polyneuropathy and cerebellar ataxia.

The question remains whether PHARC is indeed a spectrum with various incomplete phenotypes even at an older age, or whether it is a syndrome in which the clinical symptoms are variable in severity and age of onset. Remarkably, the patients described in literature suggested to have isolated retinal dystrophy arguably did not receive a thorough evaluation of clinical features to rule out neuropathy and ataxia, which makes it possible that these features have been overlooked [6, 13]. Since PHARC syndrome is a progressive disease, it could well be that, beyond a certain age, all PHARC patients may eventually manifest every symptom of the syndrome. However, with the limited available clinical information and the lack of follow-up, it is currently difficult to draw any firm conclusions.

PHARC is phenotypically related to Refsum disease, Charcot–Marie–Tooth and Usher syndrome. Therefore, these diseases should always be in the differential diagnosis of PHARC syndrome and the other way around [1, 4, 13, 22]. When patients present only with distinct auditory and ophthalmological symptoms, it is important to consider PHARC syndrome in the differential diagnosis as well.

To date, 27 different mutations in the ABHD12 gene associated with PHARC have been described, of which c.337_338delGAinsTTT is the most common (32%) and is considered a European founder mutation [1, 2]. Most mutations (including nonsense (30%), frameshift (26%), and splice-altering (11%) mutations) cause a complete loss of ABHD12 enzymatic activity. A missense mutation was identified in 26% of the patients, and in most cases, the mutation was compound heterozygous with a deleterious mutation. Only 2 patients with a homozygous missense mutation have been described. These patients did not exhibit a milder phenotype compared to patients with only loss-of-function mutations. In addition to interfamilial phenotypic variability, intrafamilial phenotypic variability also exists, indicating that there is no clear genotype–phenotype correlation within PHARC.

With the development of a mouse model, Blankman et al. (2013) discovered that lyso-PS plays a crucial role in the behavioral and cellular pathology caused by *ABHD12* silencing [3]. Although the exact mechanisms driving the pathophysiology of PHARC are not fully understood, it is hypothesized that disruption of the ABHD12 enzyme causes lipid metabolism dysregulation, leading to cellular, proinflammatory, and proapoptotic signals that could result in neuroinflammation, mast cell degranulation, lymphocyte proliferation and cell death [3, 23, 38].

Previously, suggestions for the use of lyso-PS receptors as drug targets have been proposed as these receptors are abundant in the cerebellum and are characterized by their interactions with ABHD16A, ABHD12 and the lyso-PS network [59, 60] (Fig. 2). Furthermore, additional research on ABHD16A is also important, to determine whether disruption of its ox-PS lipase activity would rescue or reverse the neurological symptoms of PHARC [60]. Lastly, inhibition of microglial phagocytosis through TLR2 blocking has been found to be sufficient to prevent inflammatory neuronal death and seems therefore relevant to the neurodegenerative character of PHARC [59].

## Conclusions

To conclude, PHARC syndrome is a rare phenotypically heterogeneous syndrome for which no effective treatment has been developed yet. We generated a clinical overview of all PHARC patients, including their mutations, described in literature so far. Clinical diagnosis is very challenging, due to the wide range of age at onset for each symptom. PHARC syndrome should always be in the differential diagnosis in patients who are clinically suspected of having Refsum disease, Charcot–Marie–Tooth disease, and Usher syndrome. We recommend adding *ABHD12* to diagnostics gene panels for hearing loss, retinal dystrophy, cataracts polyneuropathy and cerebellar ataxia. Furthermore, this overview suggests that PHARC is a syndrome in which the clinical symptoms are variable in severity and age of onset, but where most patients eventually will present with all symptoms of PHARC at a certain age. Additionally, as some subtle signs may be overlooked, patients with (suspected) PHARC should get a full clinical work-up, a neurological (with EMG and neuroimaging of the brain), an ophthalmological (with ERG) examination and audiological tests in a multidisciplinary setting involving ophthalmologists, audiologists, neurologists, and geneticists to ensure the best care.

## Data Availability

All the data generated or analyzed during this study are included in this published article.

## Abbreviations

PHARC: Polyneuropathy, hearing loss, cerebellar ataxia, retinitis pigmentosa, cataract
SNHL: Sensorineural hearing loss
ABHD12: α/β-Hydrolase domain containing 12
Lyso-PS: Lyso-phosphatidylserine
2-AG: 2-Arachidonoylglycerol
USH: Usher syndrome
EMG: Electromyography
MRI: Magnetic resonance imaging
CT: Computational tomography
CMT: Charcot–Marie–Tooth syndrome
Ox-PS: Oxidized PS
MAGL: Monoacylglycerol lipase
TLR2: Toll-like receptor 2
LPS: Lipopolysaccharide
AA: Arachidonic acid
FAAH: Fatty acid amide hydrolase
ROS: Reactive oxygen species
GPS: Glycerophosphoserine
FFA: Free fatty acid
LPCAT3: Lysophosphatidylcholine acyltransferase 3
GPCR: G-protein coupled receptor
CBR: Cannabinoid receptors
Lyso-PI: Lysophosphatidylinositol
ENT specialist: Ear-nose-throat specialist
