# Correction to: PHARC syndrome: an overview

**DOI:** 10.1186/s13023-024-03491-5

**Published:** 2025-01-08

**Authors:** Lusine Harutyunyan, Patrick Callaerts, Sascha Vermeer

**Affiliations:** 1 Laboratory for Behavioral and Developmental Genetics, Department of Human Genetics, KU Leuven, Louvain, Belgium; 2 Centre of Human Genetics, University Hospitals Leuven, Herestraat 49, Louvain, 3000 Belgium; 3 Disability Studies, Family Medicine and Population Health, University Antwerp, Antwerp, Belgium

Following publication of the original article [1], we have been notified that Figs. 2 and 3 were switched.

They are now as follows:

**Fig. 2:**
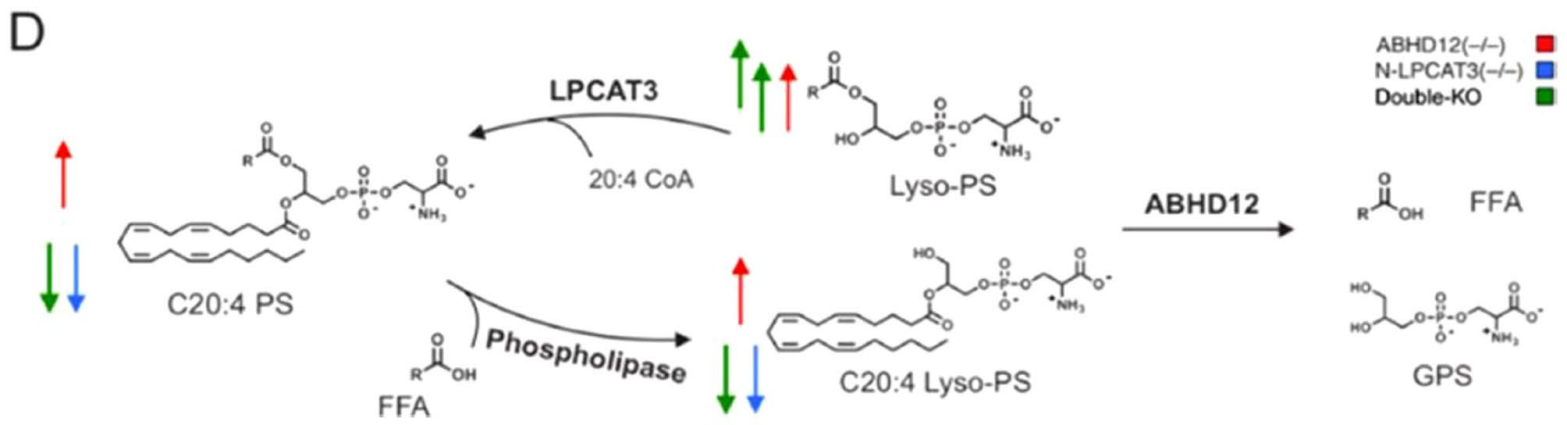
Synthesis and degradation of lyso-PS in the mouse brain. PS = phosphatidylserine; ROS = reactive-oxygen species; ox-PS = oxidized phosphatidylserine; FFA = free fatty acid; GPS = glycerophosphoserine

**Fig. 3:**
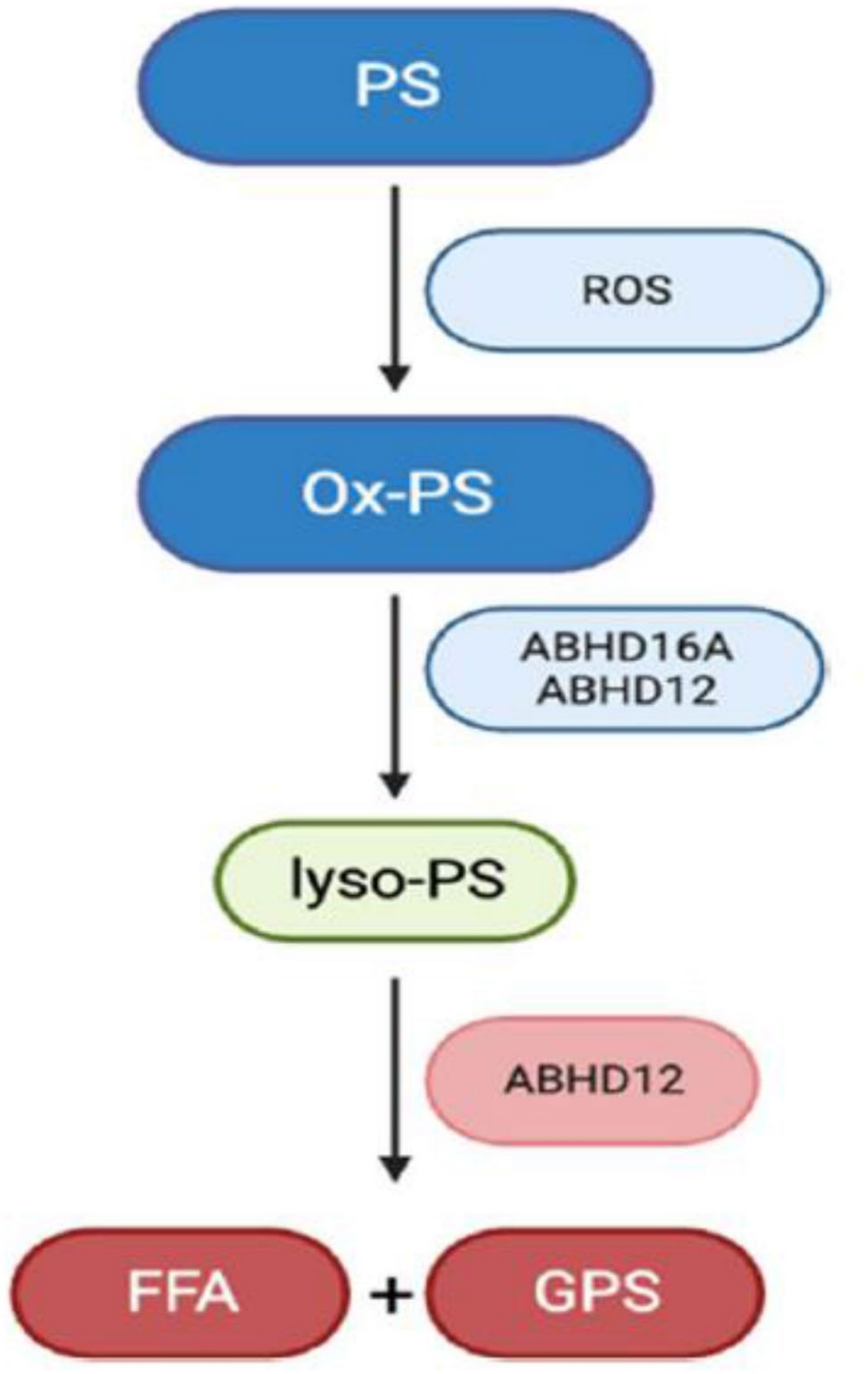
Double knockout of ABHD12 and LPCAT3 causes a strong increase in lyso-PS, but not in C20:4 PS and C20:4 lyso-PS. Metabolic pathway diagram illustrating the coordinated regulation of lyso-PS and C20:4 PS levels in the mammalian brain by ABHD12 and LPCAT3. Red arrows indicate the change in lipid content in ABHD12−/−mice, blue arrows indicate the change in lipid content in N-LPCAT3−/− mice and green arrows indicate the change in lipid content in double knockout mice. FFA = free fatty acid, GPS = glycerophosphoserine

They should be:

**Fig. 2:**
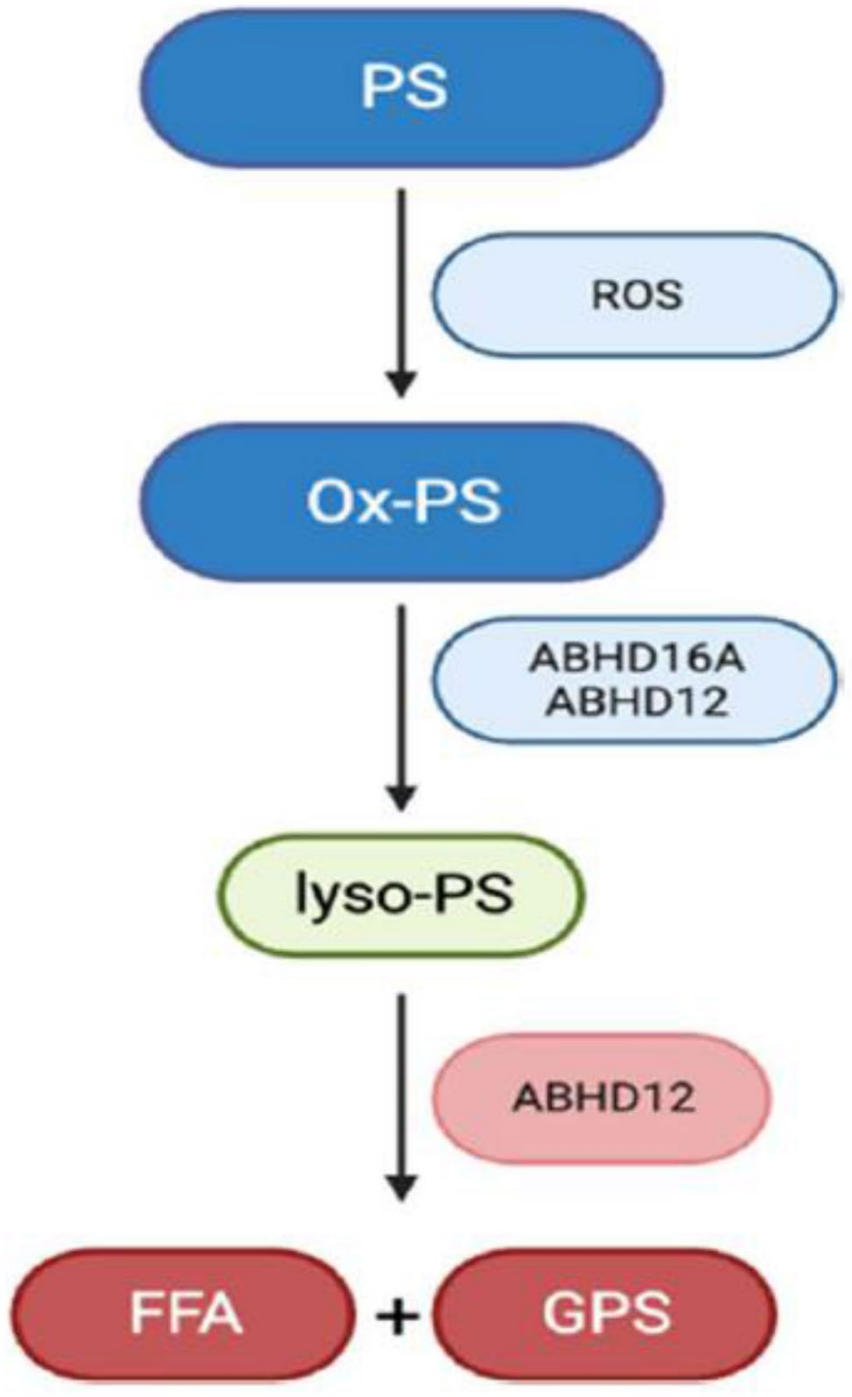
Synthesis and degradation of lyso-PS in the mouse brain. PS = phosphatidylserine; ROS = reactive-oxygen species; ox-PS = oxidized phosphatidylserine; FFA = free fatty acid; GPS = glycerophosphoserine

**Fig. 3:**
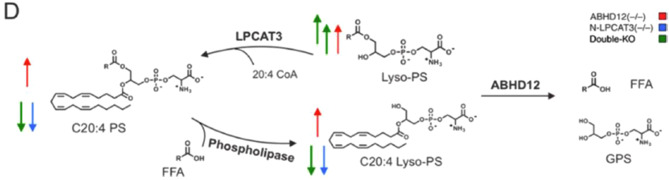
Double knockout of ABHD12 and LPCAT3 causes a strong increase in lyso-PS, but not in C20:4 PS and C20:4 lyso-PS. Metabolic pathway diagram illustrating the coordinated regulation of lyso-PS and C20:4 PS levels in the mammalian brain by ABHD12 and LPCAT3. Red arrows indicate the change in lipid content in ABHD12−/−mice, blue arrows indicate the change in lipid content in N-LPCAT3−/− mice and green arrows indicate the change in lipid content in double knockout mice. FFA = free fatty acid, GPS = glycerophosphoserine

The original article was updated.
